# Improving the quality of tuberculosis patient care in NAN hospital using the FCD NAN model

**DOI:** 10.1017/ash.2025.96

**Published:** 2025-09-03

**Authors:** Pimpaka Srijai-in

**Affiliations:** Infection Control Nurse (ICN), Nan Hospital Thailand

## Abstract

**Introduction:** During the years 2018-2019, Nan Hospitalhad tuberculosis screening rates of 69.15% and 74.24%, respectively, which were lower than the target (target > 90%). Consequently, tuberculosis patients were referred for further examination in various hospital units and treated in general rooms without proper isolation. Also, one and four healthcare personnel were diagnosed with pulmonary tuberculosis in 2018 and 2019, respectively. Additionally, the treatment success rates for pulmonary tuberculosis patients were 74.63% and 79.92%, respectively (target >85%). Therefore, to achieve these target goals, actions were taken following the national tuberculosis strategy using the FCD Nan model, which comprises of Fast screening (F), Cure (C), and do not spread (D). **Objectives:** To develop the tuberculosis patient-care system at Nan Hospital and assess the outcomes of the tuberculosis patient-care system in terms of FCD Nan model **Methods:** We used the PDSA quality improvement process as follows.

1. Plan: Planning to implement the national tuberculosis strategy including fast screening, cure, and prevention of transmission.
2. Do: Implementing the FCD Nan modelas follows.
  1. Fast screening. We used a fast-track system, enhanced screening efficiency in both OPD and IPD units, established a Line group alert pop-up and AI CXR.
  2. Cure. We adhered to treatment protocols,closely monitored cases, consulted specialists, utilized risk scoring for mortality, and nutritional alert.
  3. Do not spread. We administered 3 measures for preventing transmission including administrative, environmental, and PPE measures.
3. Study: Evaluating the FCD Nan model.
4. Action: Identifying household contacts as a high-risk group and implementing screening for all household contacts due tothe discovery of new tuberculosis patients in the household contacts.

**Results:** 1) Screening rates increased from 81.8% to 93.07% for OPD and from 70.76% to 97.29% for IPD. 2) Cure: Treatment success rate increased from 89.81% to 91.43% Do not spread: and 3) No incidence of tuberculosis among healthcare personnel. **Conclusion:** The FCD Nan model using the national tuberculosis strategy of fast screening, cure, and not spread can be used to achieve the goals of tuberculosis control.

**Acknowledgments** : This study has been partially funded by the Nursing Association for Prevention and Control of Infections (NAPCI)

